# Crystal structure of 1-{(*E*)-[(3,4-di­chloro­phen­yl)imino]­meth­yl}naphthalen-2-ol

**DOI:** 10.1107/S2056989015015959

**Published:** 2015-08-29

**Authors:** Muhammad Nawaz Tahir, Muhammad Anwar-ul-Haq, Hazoor Ahmad Shad

**Affiliations:** a Department of Physics, University of Sargodha, Sargodha, Punjab, Pakistan; b Department of Chemistry, University of Sargodha, Sargodha, Punjab, Pakistan

**Keywords:** crystal structure, naphthalen-2-ol, inversion dimers, hydrogen bonding

## Abstract

In the title compound, C_17_H_11_Cl_2_NO, the dihedral angle between the planes of the naphthalene ring system and the benzene ring is 28.88 (11)°. The main twist in the mol­ecule occurs about the N—C_b_ (b = benzene ring) bond, as indicated by the C=N—C_b_—C_b_ torsion angle of 31.0 (4)°. An intra­molecular O—H⋯N hydrogen bond closes an *S*(6) ring. In the crystal, inversion dimers linked by pairs of very weak C—H⋯O inter­actions generate *R*_2_^2^(16) loops.

## Related literature   

For related structures, see: Elmali *et al.* (1998 ▸); Pavlovic *et al.* (2002 ▸); Pierens *et al.* (2012 ▸); Yıldız *et al.* (2006 ▸); Wang *et al.* (2011 ▸).
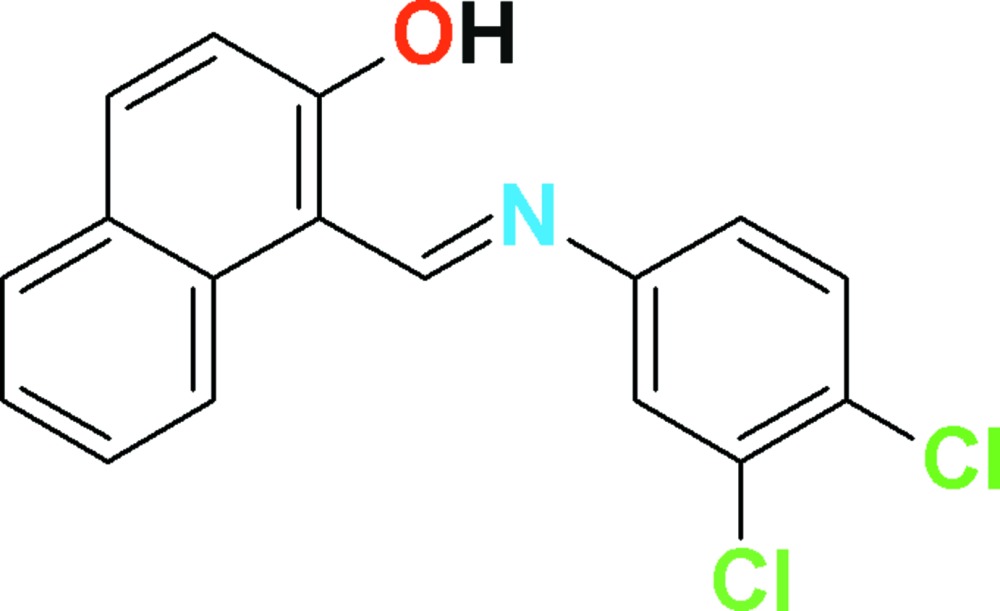


## Experimental   

### Crystal data  

C_17_H_11_Cl_2_NO
*M* *_r_* = 316.17
Monoclinic, 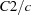

*a* = 27.075 (4) Å
*b* = 3.9284 (6) Å
*c* = 26.359 (4) Å
β = 95.287 (9)°
*V* = 2791.7 (8) Å^3^
*Z* = 8
Mo *K*α radiation
μ = 0.46 mm^−1^
*T* = 296 K
0.45 × 0.22 × 0.18 mm

### Data collection  

Bruker Kappa APEXII CCD diffractometer
Absorption correction: multi-scan (*SADABS*; Bruker, 2005 ▸) *T*_min_ = 0.823, *T*_max_ = 0.928
10968 measured reflections
3006 independent reflections
1624 reflections with *I* > 2σ(*I*)
*R*_int_ = 0.052

### Refinement  

*R*[*F* ^2^ > 2σ(*F* ^2^)] = 0.049
*wR*(*F* ^2^) = 0.113
*S* = 1.02
3006 reflections
191 parameters
H-atom parameters constrained
Δρ_max_ = 0.21 e Å^−3^
Δρ_min_ = −0.29 e Å^−3^

### 

Data collection: *APEX2* (Bruker, 2007 ▸); cell refinement: *SAINT* (Bruker, 2007 ▸); data reduction: *SAINT*; program(s) used to solve structure: *SHELXS97* (Sheldrick, 2008 ▸); program(s) used to refine structure: *SHELXL2014* (Sheldrick, 2015 ▸); molecular graphics: *ORTEP-3 for Windows* (Farrugia, 2012 ▸) and *PLATON* (Spek, 2009 ▸); software used to prepare material for publication: *WinGX* (Farrugia, 2012 ▸) and *PLATON*.

## Supplementary Material

Crystal structure: contains datablock(s) global, I. DOI: 10.1107/S2056989015015959/hb7492sup1.cif

Structure factors: contains datablock(s) I. DOI: 10.1107/S2056989015015959/hb7492Isup2.hkl

Supporting information file. DOI: 10.1107/S2056989015015959/hb7492Isup3.cml

**Fig. 1.**
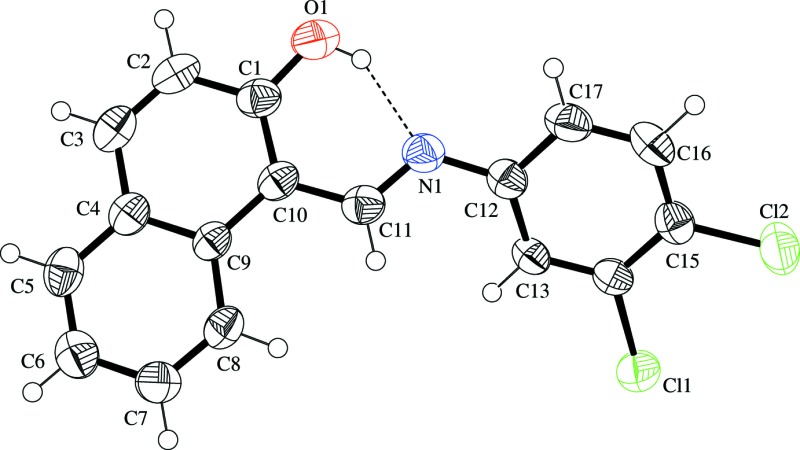
View of the title compound with displacement ellipsoids drawn at the 50% probability level. The dotted line indicates the intramolecular H-bond interaction.

. DOI: 10.1107/S2056989015015959/hb7492fig1.tif
View of the title compound with displacement ellipsoids drawn at the 50% probability level. The dotted line indicates the intra­molecular H-bond inter­action.

**Fig. 2.**
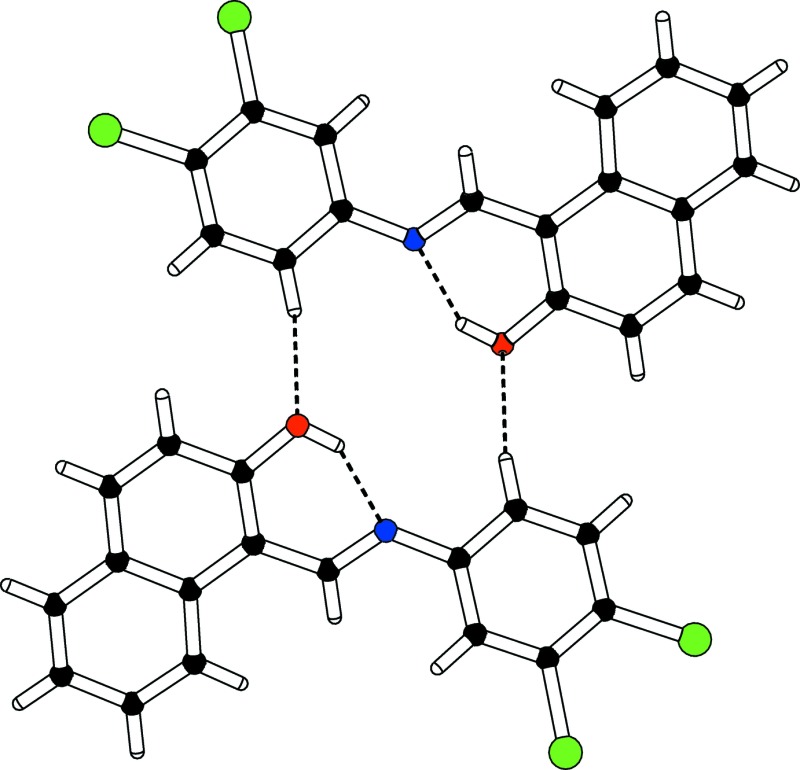
Inversion dimers in the crystal of the title compound.

. DOI: 10.1107/S2056989015015959/hb7492fig2.tif
Inversion dimers in the crystal of the title compound.

CCDC reference: 1420675

Additional supporting information:  crystallographic information; 3D view; checkCIF report

## Figures and Tables

**Table 1:** Hydrogen-bond geometry (, )

*D*H*A*	*D*H	H*A*	*D* *A*	*D*H*A*
O1H1N1	0.82	1.84	2.565(3)	147
C17H17O1^i^	0.93	2.60	3.413(3)	147

Symmetry code: (i) 
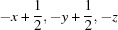
.

## supplementary crystallographic information

### S1. Comment

The crystal structures of (*E*)-1-[(2-chloro-4-nitrophenylimino)methyl]naphthalen-2-ol (Wang *et al.*, 2011), *N*-(3-chlorophenyl)-2-hydroxy-1-naphthaldimine (Pavlovic *et al.*, 2002), *N*-(2-hydroxy-1-naphthylmethylene)-2,5-dichloroaniline (Yildiz *et al.*, 2006), 1-(((4-chlorophenyl)imino)methyl)-2-naphthol (Pierens *et al.*, 2002) and *N*-(3,5-dichlorophenyl)naphthaldimine (Elmali *et al.*, 1998) have been published which are related to the title compound (I, Fig. 1).

In (I), the parts of 2-hydroxynaphthaldehyde A (C1–C11/O1) and B (N1/C12–C17/CL1/CL2) of 3,4-dichloraniline are planar with r. m. s. deviation of 0.0084 Å and 0.0111 Å, respectively. The dihedral angle between A/B is 29.00 (5)°. There exists *S* (6) ring motif due to intramolecular H-interaction of O–H···N type. The molecules are stabilized in the form of dimmers (Table 1, Fig. 2) due to C–H···O and O–H···N types of interactions and complete *R*_4_^4^(12) ring motif.

### S2. Experimental

Equimolar quantities of 3,4-dichloroaniline and 2-hydroxynaphthaldehyde were refluxed in methanol for 2 h. The solution was kept at room temperature for crystallization which afforded yellow needles after 2 h.

Melting point: 375 K

### S3. Refinement

The H-atoms were positioned geometrically (C–H = 0.93 Å, O–H = 0.82 Å) and refined as riding with *U*_iso_(H) = *xU*_eq_(C, O), where *x* = 1.5 for hydroxy and *x* = 1.2 for other H-atoms.

### Crystal data

**Table:**

C_17_H_11_Cl_2_NO	*F*(000) = 1296
*M**_r_* = 316.17	*D*_x_ = 1.502 Mg m^−^^3^
Monoclinic, *C*2/*c*	Mo *K*α radiation, λ = 0.71073 Å
*a* = 27.075 (4) Å	Cell parameters from 1624 reflections
*b* = 3.9284 (6) Å	θ = 2.3–27.0°
*c* = 26.359 (4) Å	µ = 0.46 mm^−^^1^
β = 95.287 (9)°	*T* = 296 K
*V* = 2791.7 (8) Å^3^	Needle, yellow
*Z* = 8	0.45 × 0.22 × 0.18 mm

### Data collection

**Table:**

Bruker Kappa APEXII CCD diffractometer	3006 independent reflections
Radiation source: fine-focus sealed tube	1624 reflections with *I* > 2σ(*I*)
Graphite monochromator	*R*_int_ = 0.052
Detector resolution: 7.70 pixels mm^-1^	θ_max_ = 27.0°, θ_min_ = 2.3°
ω scans	*h* = −34→34
Absorption correction: multi-scan (*SADABS*; Bruker, 2005)	*k* = −3→5
*T*_min_ = 0.823, *T*_max_ = 0.928	*l* = −33→24
10968 measured reflections	

### Refinement

**Table:**

Refinement on *F*^2^	Primary atom site location: structure-invariant direct methods
Least-squares matrix: full	Secondary atom site location: difference Fourier map
*R*[*F*^2^ > 2σ(*F*^2^)] = 0.049	Hydrogen site location: inferred from neighbouring sites
*wR*(*F*^2^) = 0.113	H-atom parameters constrained
*S* = 1.02	*w* = 1/[σ^2^(*F*_o_^2^) + (0.041*P*)^2^ + 0.8217*P*] where *P* = (*F*_o_^2^ + 2*F*_c_^2^)/3
3006 reflections	(Δ/σ)_max_ < 0.001
191 parameters	Δρ_max_ = 0.21 e Å^−^^3^
0 restraints	Δρ_min_ = −0.29 e Å^−^^3^

### Special details

**Table:**

Geometry. All e.s.d.'s (except the e.s.d. in the dihedral angle between two l.s. planes) are estimated using the full covariance matrix. The cell e.s.d.'s are taken into account individually in the estimation of e.s.d.'s in distances, angles and torsion angles; correlations between e.s.d.'s in cell parameters are only used when they are defined by crystal symmetry. An approximate (isotropic) treatment of cell e.s.d.'s is used for estimating e.s.d.'s involving l.s. planes.
Refinement. Refinement of *F*^2^ against ALL reflections. The weighted *R*-factor *wR* and goodness of fit *S* are based on *F*^2^, conventional *R*-factors *R* are based on *F*, with *F* set to zero for negative *F*^2^. The threshold expression of *F*^2^ > σ(*F*^2^) is used only for calculating *R*-factors(gt) *etc*. and is not relevant to the choice of reflections for refinement. *R*-factors based on *F*^2^ are statistically about twice as large as those based on *F*, and *R*-factors based on ALL data will be even larger.

### Fractional atomic coordinates and isotropic or equivalent isotropic displacement parameters (Å^2^)

**Table:**

	*x*	*y*	*z*	*U*_iso_*/*U*_eq_	
Cl1	−0.01545 (2)	0.1563 (2)	−0.07226 (3)	0.0583 (3)	
Cl2	0.03878 (3)	−0.1601 (2)	−0.16134 (3)	0.0657 (3)	
O1	0.23523 (6)	0.7303 (6)	0.05438 (8)	0.0661 (6)	
H1	0.2167	0.6298	0.0331	0.099*	
N1	0.15606 (7)	0.4249 (6)	0.01785 (9)	0.0480 (6)	
C1	0.21559 (9)	0.7303 (7)	0.09872 (11)	0.0473 (7)	
C2	0.24423 (10)	0.8780 (8)	0.14042 (12)	0.0550 (8)	
H2	0.2753	0.9668	0.1359	0.066*	
C3	0.22719 (10)	0.8919 (7)	0.18642 (12)	0.0514 (8)	
H3	0.2467	0.9929	0.2132	0.062*	
C4	0.18026 (9)	0.7570 (7)	0.19561 (10)	0.0434 (7)	
C5	0.16273 (10)	0.7752 (7)	0.24400 (11)	0.0510 (8)	
H5	0.1823	0.8789	0.2705	0.061*	
C6	0.11812 (11)	0.6459 (8)	0.25307 (11)	0.0568 (8)	
H6	0.1071	0.6605	0.2854	0.068*	
C7	0.08905 (10)	0.4908 (8)	0.21345 (11)	0.0555 (8)	
H7	0.0584	0.3998	0.2195	0.067*	
C8	0.10470 (9)	0.4700 (7)	0.16588 (11)	0.0465 (7)	
H8	0.0844	0.3658	0.1401	0.056*	
C9	0.15082 (8)	0.6018 (6)	0.15477 (10)	0.0380 (7)	
C10	0.16918 (9)	0.5897 (7)	0.10521 (10)	0.0407 (7)	
C11	0.14109 (9)	0.4337 (7)	0.06279 (11)	0.0446 (7)	
H11	0.1108	0.3346	0.0679	0.053*	
C12	0.12664 (9)	0.2802 (7)	−0.02362 (10)	0.0427 (7)	
C13	0.07522 (9)	0.2847 (6)	−0.02738 (10)	0.0396 (6)	
H13	0.0587	0.3810	−0.0015	0.047*	
C14	0.04871 (9)	0.1476 (7)	−0.06919 (10)	0.0390 (6)	
C15	0.07202 (10)	0.0081 (7)	−0.10842 (10)	0.0438 (7)	
C16	0.12334 (10)	0.0042 (8)	−0.10466 (11)	0.0516 (8)	
H16	0.1397	−0.0916	−0.1307	0.062*	
C17	0.15025 (10)	0.1399 (8)	−0.06306 (11)	0.0518 (8)	
H17	0.1847	0.1378	−0.0612	0.062*	

### Atomic displacement parameters (Å^2^)

**Table:**

	*U*^11^	*U*^22^	*U*^33^	*U*^12^	*U*^13^	*U*^23^
Cl1	0.0427 (4)	0.0719 (6)	0.0601 (5)	0.0001 (4)	0.0043 (3)	−0.0098 (4)
Cl2	0.0811 (5)	0.0691 (6)	0.0472 (5)	−0.0065 (4)	0.0076 (4)	−0.0140 (4)
O1	0.0435 (11)	0.0918 (19)	0.0632 (14)	−0.0072 (11)	0.0063 (10)	0.0031 (13)
N1	0.0398 (12)	0.0571 (17)	0.0472 (15)	0.0038 (11)	0.0049 (11)	0.0025 (13)
C1	0.0393 (15)	0.049 (2)	0.0532 (19)	0.0037 (13)	0.0036 (13)	0.0074 (15)
C2	0.0361 (15)	0.057 (2)	0.071 (2)	−0.0060 (13)	−0.0020 (15)	0.0009 (18)
C3	0.0463 (16)	0.045 (2)	0.060 (2)	−0.0006 (14)	−0.0104 (14)	0.0006 (16)
C4	0.0414 (15)	0.0388 (18)	0.0484 (18)	0.0069 (13)	−0.0041 (13)	0.0052 (14)
C5	0.0538 (17)	0.047 (2)	0.0503 (19)	0.0084 (14)	−0.0075 (14)	−0.0064 (15)
C6	0.0611 (19)	0.060 (2)	0.0496 (19)	0.0070 (16)	0.0051 (15)	−0.0012 (17)
C7	0.0479 (17)	0.066 (2)	0.053 (2)	−0.0032 (15)	0.0066 (14)	0.0050 (18)
C8	0.0422 (15)	0.0476 (19)	0.0476 (18)	−0.0022 (13)	−0.0066 (12)	0.0022 (15)
C9	0.0345 (13)	0.0361 (17)	0.0421 (16)	0.0040 (12)	−0.0038 (11)	0.0055 (13)
C10	0.0354 (14)	0.0375 (17)	0.0479 (17)	0.0024 (12)	−0.0036 (12)	0.0062 (14)
C11	0.0390 (14)	0.0464 (19)	0.0481 (18)	0.0039 (13)	0.0025 (13)	0.0075 (15)
C12	0.0441 (15)	0.0440 (18)	0.0403 (16)	0.0047 (13)	0.0052 (12)	0.0048 (14)
C13	0.0406 (14)	0.0441 (17)	0.0350 (15)	0.0064 (13)	0.0089 (11)	0.0011 (14)
C14	0.0418 (14)	0.0368 (17)	0.0393 (16)	0.0030 (12)	0.0083 (12)	0.0059 (14)
C15	0.0564 (17)	0.0380 (17)	0.0376 (16)	0.0009 (14)	0.0070 (13)	0.0036 (14)
C16	0.0600 (19)	0.054 (2)	0.0432 (17)	0.0122 (15)	0.0178 (14)	−0.0008 (16)
C17	0.0416 (15)	0.065 (2)	0.0500 (18)	0.0084 (15)	0.0118 (14)	0.0066 (17)

### Geometric parameters (Å, º)

**Table:**

Cl1—C14	1.732 (2)	C6—H6	0.9300
Cl2—C15	1.721 (3)	C7—C8	1.363 (4)
O1—C1	1.328 (3)	C7—H7	0.9300
O1—H1	0.8200	C8—C9	1.407 (3)
N1—C11	1.287 (3)	C8—H8	0.9300
N1—C12	1.411 (3)	C9—C10	1.441 (3)
C1—C10	1.397 (3)	C10—C11	1.431 (3)
C1—C2	1.410 (4)	C11—H11	0.9300
C2—C3	1.338 (4)	C12—C17	1.384 (4)
C2—H2	0.9300	C12—C13	1.387 (3)
C3—C4	1.418 (4)	C13—C14	1.369 (3)
C3—H3	0.9300	C13—H13	0.9300
C4—C5	1.403 (4)	C14—C15	1.374 (3)
C4—C9	1.417 (3)	C15—C16	1.384 (4)
C5—C6	1.352 (4)	C16—C17	1.367 (4)
C5—H5	0.9300	C16—H16	0.9300
C6—C7	1.389 (4)	C17—H17	0.9300
			
C1—O1—H1	109.5	C8—C9—C10	124.3 (2)
C11—N1—C12	121.4 (2)	C4—C9—C10	119.1 (2)
O1—C1—C10	123.0 (3)	C1—C10—C11	119.5 (3)
O1—C1—C2	116.7 (2)	C1—C10—C9	119.2 (2)
C10—C1—C2	120.2 (3)	C11—C10—C9	121.3 (2)
C3—C2—C1	120.8 (3)	N1—C11—C10	122.7 (2)
C3—C2—H2	119.6	N1—C11—H11	118.6
C1—C2—H2	119.6	C10—C11—H11	118.6
C2—C3—C4	121.9 (3)	C17—C12—C13	118.8 (2)
C2—C3—H3	119.0	C17—C12—N1	118.4 (2)
C4—C3—H3	119.0	C13—C12—N1	122.8 (2)
C5—C4—C9	119.9 (2)	C14—C13—C12	120.1 (2)
C5—C4—C3	121.3 (3)	C14—C13—H13	120.0
C9—C4—C3	118.8 (3)	C12—C13—H13	120.0
C6—C5—C4	121.6 (3)	C13—C14—C15	121.3 (2)
C6—C5—H5	119.2	C13—C14—Cl1	118.6 (2)
C4—C5—H5	119.2	C15—C14—Cl1	120.1 (2)
C5—C6—C7	119.1 (3)	C14—C15—C16	118.6 (2)
C5—C6—H6	120.5	C14—C15—Cl2	121.4 (2)
C7—C6—H6	120.5	C16—C15—Cl2	120.0 (2)
C8—C7—C6	121.0 (3)	C17—C16—C15	120.7 (3)
C8—C7—H7	119.5	C17—C16—H16	119.7
C6—C7—H7	119.5	C15—C16—H16	119.7
C7—C8—C9	121.8 (3)	C16—C17—C12	120.6 (2)
C7—C8—H8	119.1	C16—C17—H17	119.7
C9—C8—H8	119.1	C12—C17—H17	119.7
C8—C9—C4	116.6 (2)		
			
O1—C1—C2—C3	179.4 (3)	C4—C9—C10—C1	0.2 (4)
C10—C1—C2—C3	−1.3 (4)	C8—C9—C10—C11	−0.8 (4)
C1—C2—C3—C4	0.7 (4)	C4—C9—C10—C11	179.5 (2)
C2—C3—C4—C5	−179.6 (3)	C12—N1—C11—C10	−177.6 (2)
C2—C3—C4—C9	0.3 (4)	C1—C10—C11—N1	−2.1 (4)
C9—C4—C5—C6	0.4 (4)	C9—C10—C11—N1	178.6 (2)
C3—C4—C5—C6	−179.7 (3)	C11—N1—C12—C17	−151.6 (3)
C4—C5—C6—C7	0.1 (4)	C11—N1—C12—C13	31.0 (4)
C5—C6—C7—C8	−0.5 (4)	C17—C12—C13—C14	0.9 (4)
C6—C7—C8—C9	0.3 (4)	N1—C12—C13—C14	178.4 (2)
C7—C8—C9—C4	0.1 (4)	C12—C13—C14—C15	−1.0 (4)
C7—C8—C9—C10	−179.5 (3)	C12—C13—C14—Cl1	179.7 (2)
C5—C4—C9—C8	−0.5 (4)	C13—C14—C15—C16	0.9 (4)
C3—C4—C9—C8	179.6 (2)	Cl1—C14—C15—C16	−179.8 (2)
C5—C4—C9—C10	179.2 (2)	C13—C14—C15—Cl2	−179.3 (2)
C3—C4—C9—C10	−0.7 (4)	Cl1—C14—C15—Cl2	0.1 (3)
O1—C1—C10—C11	0.7 (4)	C14—C15—C16—C17	−0.7 (4)
C2—C1—C10—C11	−178.5 (3)	Cl2—C15—C16—C17	179.4 (2)
O1—C1—C10—C9	−179.9 (2)	C15—C16—C17—C12	0.8 (5)
C2—C1—C10—C9	0.8 (4)	C13—C12—C17—C16	−0.8 (4)
C8—C9—C10—C1	179.8 (2)	N1—C12—C17—C16	−178.4 (3)

### Hydrogen-bond geometry (Å, º)

**Table:**

*D*—H···*A*	*D*—H	H···*A*	*D*···*A*	*D*—H···*A*
O1—H1···N1	0.82	1.84	2.565 (3)	147
C17—H17···O1^i^	0.93	2.60	3.413 (3)	147

Symmetry code: (i) −*x*+1/2, −*y*+1/2, −*z*.
