# Supplementary figures and images for: Crystal structure of 1-{(E)-[(3,4-di­chloro­phen­yl)imino]­meth­yl}naphthalen-2-ol
Source: Acta Crystallogr E Crystallogr Commun. 2015 Aug 29;71(Pt 9):o696. doi: 10.1107/S2056989015015959 (PMC4555428; doi:10.1107/S2056989015015959)

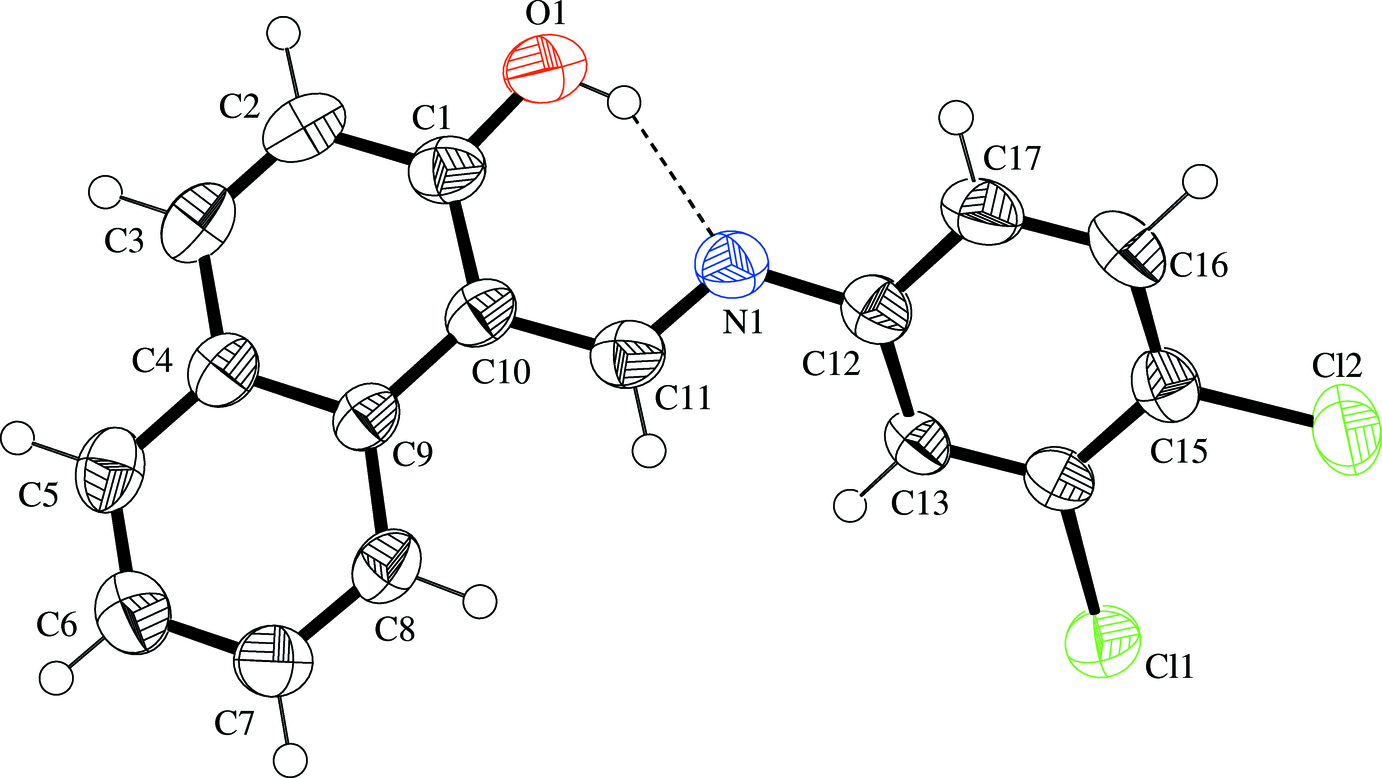

Supplement: Supplementary file 4 [file e-71-0o696-fig1.tif]

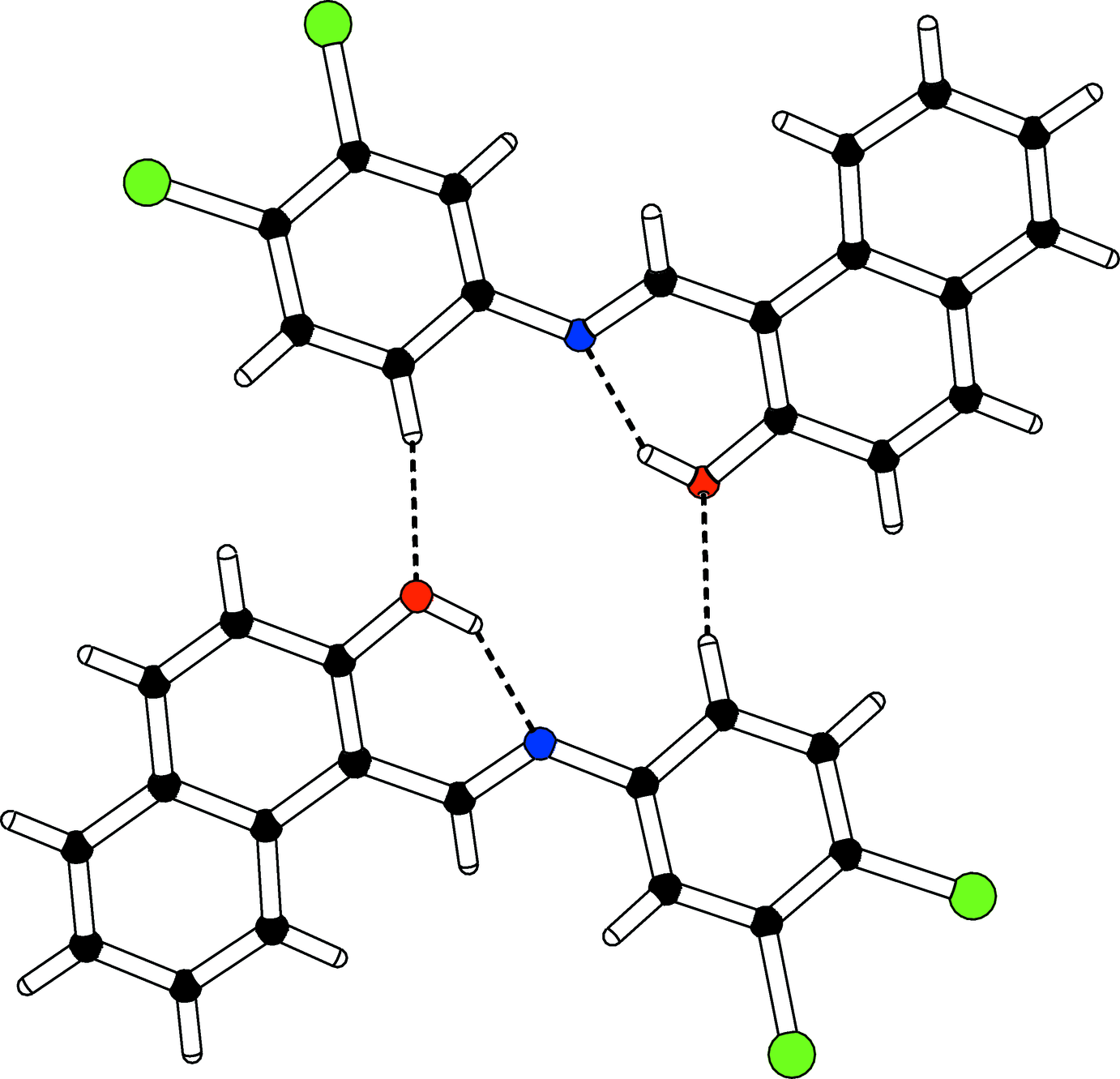

Supplement: Supplementary file 5 [file e-71-0o696-fig2.tif]
